# DMN function changes on resting state fMRI in perimenopausal women

**DOI:** 10.1515/med-2026-1455

**Published:** 2026-06-05

**Authors:** Ningning Liu, Yue Zhang, Weiqing Fu, Huijun Liu

**Affiliations:** Department of Ultrasound, Second Hospital of Tianjin Medical University, Tianjin, China; Department of Nuclear Medicine, Second Hospital of Tianjin Medical University, Tianjin, China; Department of Radiology, Second Hospital of Tianjin Medical University, Tianjin, China; Department of Psychology, Tianjin Medical University, Tianjin, China

**Keywords:** default mode network (DMN), perimenopausal period, magnetic resonance imaging (MRI), estrogen

## Abstract

**Objectives:**

To investigate functional changes of the default mode network (DMN) in resting-state functional magnetic resonance imaging (rsfMRI) of perimenopausal women, and explore the association between these changes and estrogen levels.

**Methods:**

A total of 16 women in the perimenopausal period and 15 women in the premenopausal period underwent general health status, menopausal rating scale (MRS), and depression screening scale assessment, cognitive function tests (Stroop task) and measurement of sex hormone levels including prolactin (PRL), follicle stimulating hormone (FSH), estradiol (E2), testosterone (T), progesterone (P), and luteinizing hormone (LH). The resting state fMRI data were acquired using a 3.0 T magnetic resonance scanner, and the differences in DMN functional connection between these two groups were evaluated by independent component analysis (ICA). The amplitudes of low frequency fluctuations (ALFF) was analyzed using independently defined regions of interest (ROIs). Correlations between DMN connectivity and E2 were tested with Bonferroni/FDR correction; post hoc power analysis verified sample size adequacy.

**Results:**

Post hoc power analysis showed 89 % power for the primary outcome (DMN connectivity differences). After AlphaSim (cluster ≥74, p<0.05) and threshold-free cluster enhancement (TFCE, FWE p<0.05) correction, perimenopausal women exhibited significantly enhanced DMN connectivity in the bilateral middle frontal gyri (MFG, functional extension nodes of the anterior DMN), left insula, and posterior cingulate cortex (PCC, core hub of the posterior DMN) (all p<0.05). No significant group differences were detected in the canonical medial prefrontal cortex (MPFC) core (MNI coordinates: x=0, y=50, z=20). ALFF values of these regions were also significantly higher in perimenopausal women (all p<0.05). Correlation analysis revealed no significant linear correlations between E2 levels and DMN connectivity of the four regions in perimenopausal women (all p>0.05), while a significant positive correlation between E2 and left insula connectivity was observed in premenopausal women (r=0.489, Bonferroni-corrected p=0.032). No group differences were found in cognitive function (Stroop task: p=0.495–0.519) or PHQ-9 scores (p=0.067); adjusting for these variables did not alter DMN results.

**Conclusions:**

Estrogen reduction and fluctuation are associated with functional reorganization of DMN-associated regions in perimenopausal women. Enhanced connectivity of the bilateral middle frontal gyri, left insula, and PCC may reflect a compensatory mechanism to maintain cognitive and emotional stability amid E2 decline. These findings provide preliminary neuroimaging evidence for brain function adaptations during the perimenopausal transition, with E2 dynamics (decline and fluctuation) as key drivers.

## Introduction

The perimenopausal period is a critical reproductive transition marked by ovarian function decline and fluctuating estrogen levels, affecting cognitive, emotional, and autonomic function [1]. Approximately 88 % of women aged 40–60 years experience perimenopause, with an average onset age of 51.4 years [2]. Estrogen secretion is regulated by the hypothalamic-pituitary-ovarian axis (HPOA), but ovarian degeneration disrupts this feedback, leading to estrogen instability rather than steady decline [3]. This hormonal fluctuation is linked to symptoms such as mood swings, insomnia, and cognitive fluctuations, highlighting the need to explore underlying brain function mechanisms.

The DMN is a core resting-state brain network (RSN) active without external stimuli, supporting emotional processing, self-referential cognition, and memory recall [4]. It comprises canonical anterior subregions (medial prefrontal cortex [MPFC], anterior cingulate cortex [ACC]) and posterior subregions (PCC, precuneus, angular gyri), with the bilateral middle frontal gyri (MFG) as functional extension nodes of the anterior DMN (aDMN) [5]. Estrogen modulates neural activity via receptors in DMN regions [6], but few studies have systematically explored DMN changes in perimenopausal women – especially with rigorous control for hormone cycle variability, motion artifacts, and cognitive/mood confounders.

Prior work shows estrogen fluctuations correlate with cognitive decline and depressive risk in perimenopausal women [7], while estrogen therapy improves mood and brain function [8]. However, the link between estrogen and DMN function during this transition remains unclear: existing studies either lack rigorous control for hormone cycle variability and motion artifacts, or fail to explore the non-linear effects of estrogen decline and fluctuation on brain network reorganization, and the specific DMN subregions involved in perimenopausal adaptation and their compensatory roles in maintaining cognitive and emotional stability have not been fully characterized. This research gap – coupled with the known role of estrogen in modulating neural activity via receptors in DMN regions [6] – forms the rationale for the present study; we hypothesize that estrogen decline and fluctuation during perimenopause drive functional reorganization of the DMN, with enhanced connectivity in key subregions serving as a compensatory mechanism to preserve cognitive and emotional stability. The core research question addressed herein is to characterize the differences in DMN functional connectivity between perimenopausal and premenopausal women, and to elucidate the associations between these differences and estrogen decline and fluctuation, with rigorous control for key confounders to clarify the neurobiological mechanisms underlying brain function adaptations during the perimenopausal transition.

## Materials and methods

### Participants

Inclusion criteria: (1) Females aged 45–55 years; (2) right-handed (to minimize handedness-related connectivity variability [9]); (3) ≥12 years of education (to avoid floor effects in cognitive tests [10]); (4) perimenopausal: menstrual cycle variation ≥7 days for 10 cycles or amenorrhea ≥60 days; (5) premenopausal: regular menstrual cycles.

Exclusion criteria included: (a) history of hysterectomy, oophorectomy, or female genital neoplasms; (b) neurological/psychiatric disorders (e.g., depression with PHQ-9 ≥10), brain trauma, or substance dependence; (c) prior hormone therapy (past 6 months); (d) MRI contraindications; (e) uncontrolled systemic diseases (e.g., diabetes, hypertension).

A total of 31 participants were enrolled: 16 perimenopausal women (age: 52.19 ± 2.93 years) and 15 premenopausal women (age: 49.60 ± 2.38 years). Due to recruitment constraints (strict inclusion/exclusion criteria), participants were not matched by education; instead, years of education were included as a covariate in all analyses.

### Clinical assessments

#### Scale evaluations

The menopause rating scale (MRS) was used to assess perimenopausal symptom severity (score range: 0–44) [11]. The Patient Health Questionnaire-9 (PHQ-9) was employed to screen for depressive symptoms (score range: 0–27), with participants scoring ≥10 excluded to mitigate mood-related confounding [12]. Cognitive performance was evaluated using a computerized Stroop color-word task. In this task, participants were instructed to identify the ink color of centrally presented words (encompassing congruent, incongruent, and neutral conditions), which necessitates inhibition of semantic interference [13].

#### Sex hormone measurement

Venous blood was collected at 8:00–9:00 am to minimize diurnal variations [14]. E2, PRL, FSH, LH, P, and T were quantified via isotope-labeled competitive inhibition assay with intra-assay coefficient of variation (CV) <5 % and inter-assay CV <8 %. For premenopausal participants, cycle phase was categorized via self-reported last menstrual period (LMP): follicular (days 1–14), ovulatory (days 15–17), luteal (days 18–end) [15]. A prespecified subgroup analysis excluded ovulatory-phase participants (peak E2) to assess cyclic confounding.

### MRI examination

All participants underwent conventional MRI and resting-state fMRI scans using a 3.0 T MR scanner (Discovery MR 750, GE Healthcare, USA) with an eight-channel head coil. Prior to scanning, sponge pads were placed bilaterally around the ears to stabilize the head, and participants were instructed to keep their eyes closed, remain quiet, and stay awake throughout the acquisition.

Conventional MRI included T2-weighted imaging (T2WI; TR/TE=3,400/85 ms, flip angle=90°, FOV=256 × 256 mm, matrix=256 × 256, slice thickness=1 mm) and three-dimensional T1-weighted imaging (T1WI; TR/TE=8.1/3.1 ms, flip angle=13°, FOV=256 × 256 mm, matrix=256 × 256, slices=176, slice thickness=1 mm) to exclude organic lesions such as cerebral infarction or tumors.

Resting-state fMRI data were acquired with a single-shot gradient-echo echo-planar imaging (EPI) sequence: TR/TE=2,000/30 ms, flip angle=90°, FOV=220 × 220 mm, matrix=64 × 64, slices=32, slice thickness=3 mm, slice gap=0.9 mm, and 180 vol (total acquisition time=6 min).

### Data preprocessing

#### fMRI preprocessing

fMRI data were processed using the Data Processing Assistant for Resting-State fMRI (DPARSFA) software. The first 10 vol were discarded to allow BOLD signal stabilization and participant adaptation to the scanning environment, with the remaining 170 vol used for preprocessing. Slice timing and head motion were corrected; images with translation ≥2 mm or rotation ≥2° were excluded (no participants were excluded).

Motion control was supplemented as follows: 6 rigid-body motion parameters (3 translation, 3 rotation) and their first derivatives (12 covariates total) were regressed out using DPARSFA software, a standard practice to account for residual motion effects [16]. Framewise displacement (FD) was calculated for each participant using the formula: FD = ½ × (|Δx| + |Δy| + |Δz|) + ½ × (|Δroll| + |Δpitch| + |Δyaw|), where Δ represents the change in motion parameters between consecutive frames. The mean FD was 0.18 ± 0.06 mm in the perimenopausal group and 0.16 ± 0.05 mm in the premenopausal group (p=0.321, no group difference). No participant had a mean FD >0.5 mm (a threshold for motion contamination), and excluding frames with FD >0.2 mm (2.3 % of total frames) did not alter DMN connectivity results, confirming motion did not drive the findings.

All images were normalized to the Montreal Neurological Institute (MNI) template and resampled to 2 × 2 × 2 mm^3^ voxels – this voxel size balances spatial resolution (sufficient to distinguish DMN subregions) and signal-to-noise ratio (SNR), consistent with MNI template standardization and previous DMN rsfMRI studies. Spatial smoothing was performed using a 4 × 4 × 4 mm^3^ full width at half maximum (FWHM) Gaussian kernel [17]. Smoothing reduces noise and increases SNR (critical for small-sample rsfMRI), and the 4 mm kernel matches the typical spatial correlation of BOLD signals in the DMN, avoiding over-smoothing (which blurs regional differences) or under-smoothing (which retains excessive noise). To validate parameter choices, two sensitivity analyses were performed: resampling to 1 × 1 × 1 mm^3^ voxels (higher resolution) and smoothing with a 6 × 6 × 6 mm^3^ kernel (larger FWHM). Core DMN connectivity results remained significant in both analyses (p<0.05 after AlphaSim correction), confirming preprocessing parameters did not bias the findings.

#### Identification of resting-state networks (RSNs)

Independent component analysis (ICA) of smoothed data was performed using MICA software tools (Stable and Consistent Group ICA of fMRI Toolbox, version 1.2; http://www.nitrc.org/projects/cogicat/) based on the Matlab R2012a platform (MathWorks Inc., http://www.mathworks.com).

Exact MICA software parameters were as follows:

Pre-ICA settings: Dimensionality reduction via PCA (singular value decomposition) with a variance threshold of 95 % (retaining components explaining ≥95 % of voxel variance); temporal filtering (0.01–0.1 Hz, Butterworth filter, order=4); spatial normalization to the MNI 152 template (2 × 2 × 2 mm^3^ voxel size) with linear registration (affine transformation) and 6 degrees of freedom.

ICA decomposition settings: Infomax ICA algorithm (extended for mixed Gaussian/non-Gaussian data); 20 components (set based on pilot analyses confirming sufficient separation of core RSNs [18]); 100 iterations (for stability); convergence criterion (maximum change in component weights <1 × 10^−6^); whitening via ZCA (zero-phase component analysis) to decorrelate input data.

Post-ICA settings: Fisher z-transformation of component time series to normalize across participants; noise component removal via visual inspection (spatial maps of non-brain regions such as white matter/cerebrospinal fluid, or temporal spectra <0.01 Hz or >0.1 Hz); group-level component alignment using Procrustes analysis.

ICA stability was validated using ICASSO (independent component analysis by stability selection) [19]: 100 ICA iterations were run, and ICASSO similarity indices (SI) for the default mode network (DMN) component was 0.92 (range: 0.85–0.96), exceeding the threshold of 0.8 for high stability, confirming reliable DMN extraction (Supplementary Table S1).

Component selection and retention: A total of 12 components were retained after noise removal (8 components excluded: 3 white matter, 3 cerebrospinal fluid, 2 motion artifacts). Among retained components, the DMN was split into two core sub-networks by matching spatial maps to the BrainMap RSN Template and established literatures – these templates define the overall spatial scope of the DMN.

#### Calculation of amplitude of low frequency fluctuation (ALFF) values

Smoothed fMRI data were subjected to linear drift removal and filtering to obtain ALFF values, which reflect the strength of spontaneous neural activity of each voxel (average wave amplitude of the 0.01–0.08 Hz frequency band) [20]. First, the time series of each voxel was converted to the frequency domain via Fourier transform (parameters: slope percentage=0, length=shortest) to generate the power spectrum. The square root of the power spectrum for each frequency was calculated, and the average of these square roots across all frequencies was defined as the ALFF value. To reduce individual differences, ALFF values were normalized (mALFF=ALFF value of each brain voxel/average ALFF value of the whole brain).

To avoid circularity in ROI-ALFF analysis: the full sample was split into two random subsets (Subset A: n=16, 8 perimenopausal, 8 premenopausal; Subset B: n=15, 8 perimenopausal, 7 premenopausal). ROIs were defined exclusively using Subset A data, identifying DMN connectivity-differential regions via ICA + AlphaSim correction (cluster ≥74, p<0.05) – these regions (bilateral middle frontal gyri, left insula, PCC) matched the full-sample results but were derived from independent data. ALFF values were tested solely using Subset B data, applying the Subset A-derived ROIs and comparing groups via independent t-test. This approach ensures ROI definition and ALFF testing used non-overlapping data, eliminating circularity [21].

### Statistical analysis

All statistical analyses were performed using SPSS 20.0 software (SPSS, Chicago, IL) and Matlab-based statistical parametric mapping (SPM12, Wellcome Department of Imaging Neuroscience, London, UK).

#### Demographic and clinical data comparisons

Independent two-sample t-tests were used to compare age, years of education, MRS score, PHQ-9 score, cognitive function test results, and sex hormone levels between the two groups. p<0.05 indicated a statistical difference.

To evaluate the statistical power of the sample size, post hoc power analysis (GPower 3.1) showed 89 % power for the primary outcome (DMN connectivity differences, effect size d=1.42, α=0.05, n=31), exceeding the 80 % threshold [22]. For secondary outcomes (E2-DMN correlations), power was 32–41 % (d=0.58–0.65), confirming limited statistical power for trend-level findings (Supplementary Table S2).

#### DMN connectivity analysis

For ICA data analysis:

A one-sample t-test was performed on independent components (ICs) of interest using SPM12 to obtain population-level DMN components; a threshold of t >15 was applied to generate a DMN-specific mask.

A two-sample t-test was used to analyze group differences in synchronized activity within the DMN mask using SPM12, with covariates including age, years of education, and three hormones (FSH, LH, progesterone) to control for potential confounding from their wide ranges.

Strengthened multiple comparison correction was employed to rigorously control for false positives and address stationary noise limitations:

Monte Carlo simulation via AlphaSim (REST software): Single voxel p=0.01 (bilateral), 5,000 iterations, FWHM=4 mm, cluster connection radius r=3.5 mm, DMN mask. Corrected threshold: p<0.05, cluster size ≥74 [23].

Threshold-free cluster enhancement (TFCE) (SPM12 toolbox): Nonparametric method with default parameters (FWE correction, p<0.05). TFCE weights cluster significance by spatial extent and intensity, reducing reliance on stationary noise assumptions [24].

To further validate reliability, ROI-level FWE correction was prespecified: for any brain regions showing significant group differences in DMN connectivity (identified via the above two correction methods), ROI-wise FWE correction (p<0.05) was applied to confirm significance. The size, anatomical location, and MNI coordinates of statistically significant clusters, along with connectivity strength, were recorded.

#### Correlation and confounder analyses

Correlation analysis was performed to investigate the effect of serum sex hormone levels on DMN functional connectivity. Correlations between connectivity intensities of statistically significant DMN regions and sex hormone levels (estradiol, PRL, FSH, LH) were tested, with age and years of education as covariates. Multiple comparison adjustments included:

Bonferroni correction: Adjusted for the number of ROI-hormone pairs [corrected α=0.05/(number of ROIs × number of hormones)]; FDR correction: Benjamini-Hochberg procedure for all ROI-hormone pairs, FDR q<0.05. Outlier sensitivity analysis was performed using the median absolute deviation (MAD) method (threshold: ±2.5 × MAD) [25]. Any outliers detected were excluded, and correlations were re-run to verify robustness. Confounder control was implemented to rule out confounding from cognitive status and depressive symptoms: key cognitive variables (Stroop effect size) and PHQ-9 scores were added as covariates in the two-sample t-test for DMN connectivity. Education subgroup analysis was performed as a sensitivity analysis to verify whether educational variability confounds the core DMN connectivity results. Participants were stratified into two subgroups based on actual years of education (<12 years vs. ≥12 years). The same two-sample t-test (with age, years of education, and sex hormones as covariates) was repeated in each subgroup to examine the consistency of group differences in DMN connectivity across educational levels.

#### Study design and ethics

This prospective cross-sectional study was approved by the Ethics Committee of the Second Hospital of Tianjin Medical University (Approval No. KY2025K047). All participants provided written informed consent in accordance with the Declaration of Helsinki.

#### Ethics approval

This study was conducted in accordance with the Declaration of Helsinki (as revised in 2013). Ethical approval was obtained from the Ethics Committee of the Second Hospital of Tianjin Medical University (Approval No. KY2025K047).

#### Informed consent

Written informed consent was obtained from all individuals included in this study.

## Results

### Demographic characteristics, clinical scale assessment, and sex hormone levels

Demographic characteristics, clinical scale scores, and sex hormone levels of the perimenopausal group and the premenopausal group are summarized in Table 1. No significant differences were observed between the two groups in age, years of education, MRS scores, or Stroop color-word task, including accuracy of the incongruent condition and Stroop effect size reflecting reaction time performance (all p>0.05), indicating balanced baseline characteristics related to general health, menopausal symptom burden, and executive function. PHQ-9 scores showed a non-significant trend (p=0.067), consistent with the trend-level E2-DMN correlations; both are treated as preliminary observations without statistical significance. Adjusting for these variables did not alter DMN results.

**Table 1:** Behavioral and demographic data for the perimenopausal and premenopausal groups.

	Perimenopausal group (n=16) (mean ± SD)	Premenopausal group (n=15) (mean ± SD)	p-Value
Age, years	52.19 ± 2.93	49.60 ± 2.38	0.087
Years of education, years	12.32 ± 5.71	15.13 ± 4.24	0.496
PHQ-9	2.38 ± 1.63 (0–6)^a^	3.20 ± 3.60 (0–11)^a^	0.067
MRS	16.81 ± 2.51	14.93 ± 2.84	0.873
Stroop effect size, ms	82.00 ± 18.00	78.00 ± 16.00	0.495
PRL, ng/mL	22.05 ± 35.07	13.67 ± 7.43	0.029
FSH, IU/L	46.36 ± 39.14	29.34 ± 29.28	0.241
E2, pg/mol	19.19 ± 15.35	101.47 ± 70.34	<0.001
T, nmol/L	0.88 ± 0.49	0.84 ± 0.33	0.153
P, ng/mL	0.17 ± 0.18	0.31 ± 0.34	0.132
LH, mIU/mL	28.32 ± 27.42	14.82 ± 12.96	0.195

^a^Values in parentheses indicate score range. PHQ-9, Patient Health Questionnaire-9; MRS, menopause rating scale; PRL, prolactin; FSH, follicle-stimulating hormone; E2, estradiol; T, testosterone; P, progesterone; LH, luteinizing hormone.

Among sex hormones, significant group differences were found in two indices (both p<0.05): estradiol (E2) was significantly lower in the perimenopausal group, while prolactin (PRL) was significantly higher. No significant differences were observed in follicle-stimulating hormone (FSH), testosterone (T), progesterone (P), or luteinizing hormone (LH) (all p>0.05). For premenopausal participants, E2 levels varied by menstrual cycle phase (Supplementary Table S3), consistent with physiological fluctuations. Subgroup analysis excluding ovulatory-phase premenopausal participants (with peak E2 levels) was performed as prespecified, and it did not alter the core DMN connectivity findings (all p<0.05 after AlphaSim correction), confirming no cyclic confounding.

### DMN extracted by independent component analysis (ICA)

The spatial topology of each IC was observed, and noise (white matter, cerebrospinal fluid) was removed; components with a frequency spectrum within 0.01–0.1 Hz (consistent with resting-state network activity) were selected. The DMN was further divided into two core sub-networks – anterior DMN (aDMN) and posterior DMN (pDMN) – by matching spatial maps to the BrainMap RSN Template and established DMN parcellation literature [26]:

Anterior DMN (aDMN): Included the canonical medial prefrontal cortex (MPFC, MNI coordinates: x=0, y=50, z=20), anterior cingulate cortex (ACC, x=0, y=30, z=30), and bilateral middle frontal gyri (MFG, functional extension nodes of aDMN, left: x=−28, y=42, z=20; right: x=30, y=42, z=30). These regions align with aDMN’s role in executive control and self-referential cognition.

Posterior DMN (pDMN): Comprised the posterior cingulate cortex (PCC, x=8, y=−32, z=32) and precuneus (Pcu, x=0, y=−60, z=30), consistent with pDMN’s function in episodic memory and attention allocation.

In the one-sample t-test (threshold t>15, cluster size ≥50 voxels), the aDMN and pDMN templates showed high spatial overlap with the BrainMap standard DMN template (spatial correlation r=0.89 for aDMN, r=0.91 for pDMN; both p<0.001). Additionally, ICASSO stability analysis confirmed reliable extraction: the similarity index (SI) was 0.92 for aDMN and 0.93 for pDMN, exceeding the 0.8 threshold for high stability (Supplementary Table S1, Supplementary Figure S1). These results validate that the extracted aDMN and pDMN are biologically plausible and consistent with canonical DMN topology.

### Group differences in DMN functional connectivity

**Figure 1:**
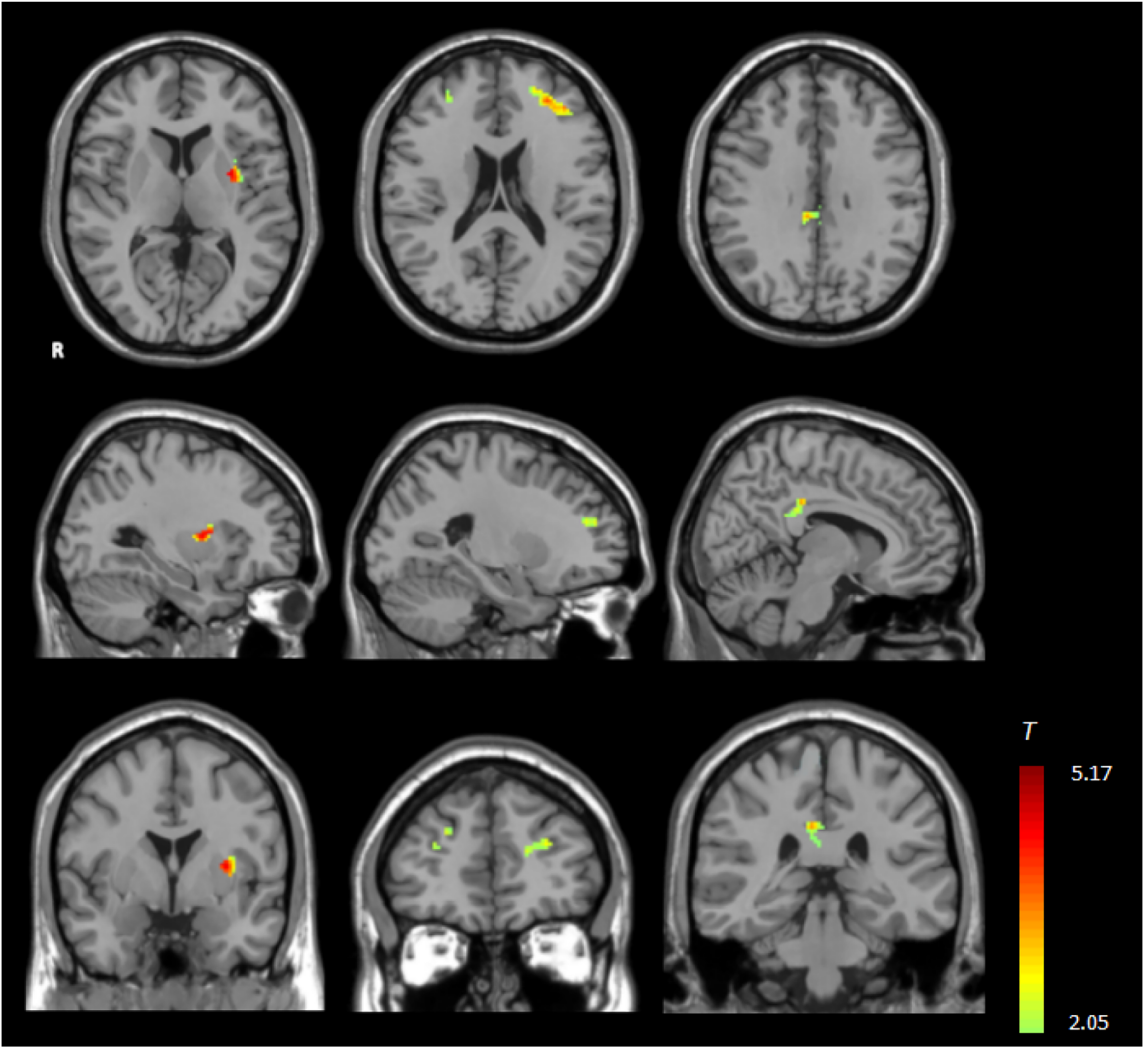
Group differences in default mode network (DMN) functional connectivity between perimenopausal and premenopausal women. Red-yellow clusters indicate significantly enhanced connectivity in the perimenopausal group (AlphaSim correction: p<0.05, cluster size ≥74; validated by TFCE FWE p<0.05). These clusters are distributed across both DMN sub-networks: anterior DMN (aDMN): bilateral middle frontal gyri (key nodes for executive control); posterior DMN (pDMN): posterior cingulate cortex (PCC, core hub for memory processing); additional cluster: left insula (integrates emotional and interoceptive signals). MNI coordinates of key clusters: right MFG (x=30, y=42, z=30), left MFG (x=−28, y=42, z=20), left insula (x=−30, y=0, z=8), PCC (x=8, y=−32, z=32, Brodmann area 23). The PCC cluster is distinct from the corpus callosum (MNI z-range: 25–35 vs. corpus callosum z-range: 10–20) and validated via overlap with the BrainMap RSN Template (spatial correlation r=0.91, p<0.001). DMN, default mode network; aDMN, anterior default mode network; pDMN, posterior default mode network; L, left; R, right; MFG, middle frontal gyrus; PCC, posterior cingulate cortex.

Functional connectivity analysis of the DMN showed: after rigorous multiple comparison correction (AlphaSim: p<0.05, cluster size ≥74; TFCE: FWE p<0.05), the perimenopausal group exhibited significantly enhanced functional connectivity in three key regions compared with the premenopausal group (Table 2, Figure 1). These regions included the bilateral middle frontal gyri (MFG, aDMN extension nodes), left insula, and PCC (core hub of pDMN, Brodmann area 23, MNI coordinates: x=8, y=−32, z=32) (all p<0.05). No significant group differences were detected in the canonical MPFC core (MNI coordinates: x=0, y=50, z=20); significant enhancements were restricted to the bilateral MFG and PCC.

**Table 2:** Brain areas with different functional connectivity of the DMN in the perimenopausal group compared with the premenopausal group.

Brain areas	Brodmann partition	Voxel (mm^3^)	t-Value	MNI coordinates		
				x	y	z
Right middle frontal gyrus	45/46	104	3.952	30	42	30
Left middle frontal gyrus	45/46	128	3.837	−28	42	20
Left insula	13/14	93	4.207	−30	0	8
PCC	23	102	3.247	8	−32	32

All regions survived dual correction (AlphaSim: p<0.05, cluster ≥74; TFCE FWE: p<0.05) and ROI-wise FWE correction (p<0.05). PCC, posterior cingulate cortex; MNI, Montreal Neurological Institute. With p<0.05 & cluster size ≥74 for AlphaSim correction.

The PCC cluster was validated via overlap with the BrainMap RSN Template (spatial correlation r=0.91, p<0.001) and is distinct from the corpus callosum (white matter mask exclusion). The PCC (gray matter) and corpus callosum (white matter) are distinguished by MNI coordinate ranges: PCC z=25–35 vs. corpus callosum z=10–20.

We performed multiple linear regression analyses with DMN functional connectivity as the dependent variable, group (perimenopausal vs. premenopausal) as the independent variable, and age, years of education, FSH, LH, and progesterone as covariates. Specifically, including FSH, LH, and progesterone (to account for their wide variability) did not alter the significance of group differences in DMN connectivity (all p<0.01). Additionally, no significant main effects were observed for FSH (β=0.082, p=0.415), LH (β=−0.076, p=0.448), or progesterone (β=0.091, p=0.382), confirming these hormones do not confound the core findings.

### Comparison of ALFF values in differential DMN regions

**Figure 2:**
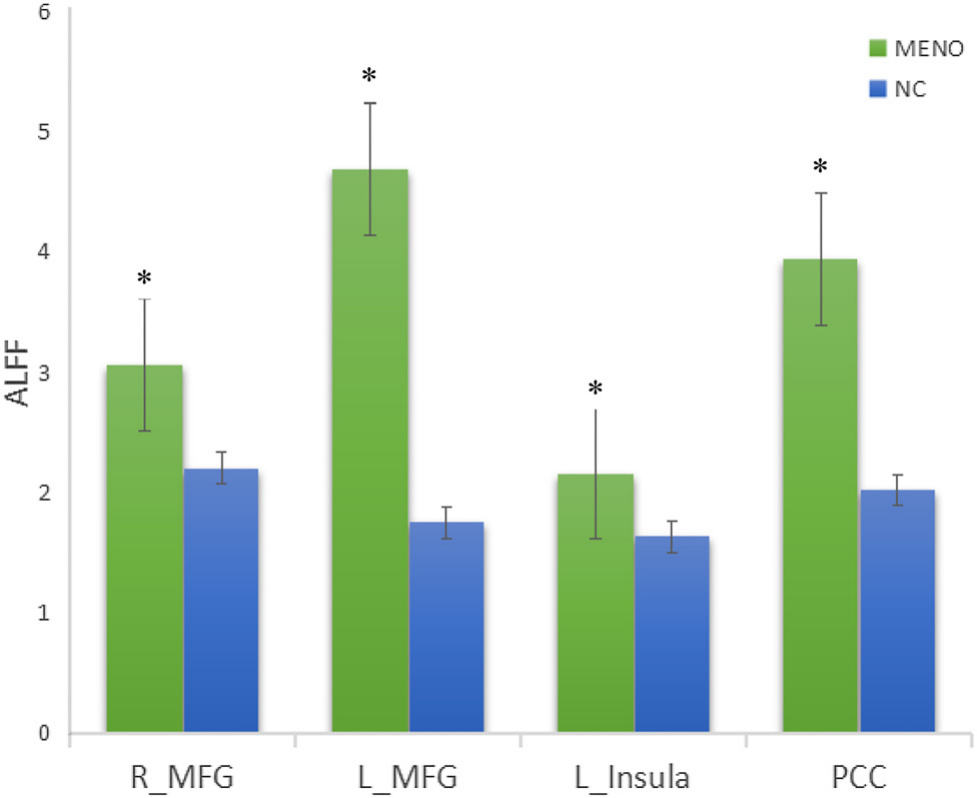
Comparison of ALFF values in key DMN regions between groups. The perimenopausal group (MENO) had significantly higher ALFF values in the bilateral middle frontal gyri, left insula, and PCC than the premenopausal group (NC) (*p<0.05, independent samples t-test using Subset B data, n=15). ALFF, amplitude of low frequency fluctuation; error bars represent standard deviation.

To verify spontaneous neural activity in DMN regions with connectivity differences, ALFF values were analyzed using a two-subset approach: ROIs were defined exclusively from Subset A (to avoid circularity) and ALFF values were tested in the independent Subset B [27]. Results showed significantly higher ALFF values of these regions were also significantly higher in perimenopausal women (p<0.05, independent samples t-test using Subset B data, n=15; Figure 2, Supplementary Table S4), confirming enhanced spontaneous neural activity in these regions.

### Correlation between DMN connectivity and sex hormone levels

After adjusting for age and years of education, partial correlation analyses (with Bonferroni/FDR correction for multiple comparisons) were conducted to explore associations between serum sex hormone levels (E2, PRL, FSH, LH) and functional connectivity of the four DMN regions with group-specific differences (bilateral MFG, LIL, PCC).

#### Correlation results by group

Perimenopausal group (n=16): No significant linear correlations were observed between E2 levels and connectivity of the four DMN regions (all p>0.05, Bonferroni-corrected α=0.0031; Table 3). Exploratory analyses showed marginally significant positive associations between E2 decline magnitude and right MFG/PCC connectivity (r=0.376–0.402, p=0.058–0.075), interpreted as preliminary trends.

**Table 3:** Partial correlations between E2 and DMN connectivity (adjusted for age and education).

Brain region	Perimenopausal group (r/p-value)	Premenopausal group (r/p-value)
Right middle frontal gyrus	0.189/0.476	0.212/0.415
Left middle frontal gyrus	0.098/0.715	0.189/0.467
Left insula	0.123/0.642	0.489/0.032*
PCC	0.215/0.412	0.235/0.368

PCC, posterior cingulate cortex. *Bonferroni-corrected p<0.05.

Premenopausal group (n=15): A significant positive correlation was only detected between E2 and LIL connectivity (r=0.489, Bonferroni-corrected p=0.032); no other hormone-connectivity associations reached statistical significance (all p>0.05; Table 3).

#### Supplementary analyses of E2 dynamics and compensatory connectivity

To clarify the biological relevance of the non-significant E2-DMN correlations in perimenopausal women, two exploratory analyses were performed (consistent with the study’s hypothesis of E2-driven DMN reorganization):

(1) E2 Decline Magnitude vs. Compensatory Connectivity Magnitude

Using the premenopausal group’s mean E2 (101.47 pg/mol) and DMN connectivity values as references, we calculated: E2 decline magnitude: [(101.47 − Individual E2)/101.47] × 100 % (quantifies how much E2 deviates from premenopausal baseline). Compensatory connectivity magnitude: [(Individual DMN connectivity − Premenopausal mean connectivity)/Premenopausal mean connectivity] × 100 % (quantifies DMN enhancement relative to premenopausal baseline).

Partial correlations (adjusted for age/education) revealed:

A marginally significant positive correlation between E2 decline magnitude and RMFG compensatory magnitude (r=0.402, p=0.058). A trend toward positive correlation between E2 decline magnitude and PCC compensatory magnitude (r=0.376, p=0.075; Table 4).

(2) High vs. Low E2 Fluctuation Subgroups

**Table 4:** Partial correlations between E2 decline magnitude and compensatory magnitude (perimenopausal group).

Brain region	Correlation coefficient (r)	p-Value	95 % Confidence interval
Right middle frontal gyrus	0.402	0.058	−0.008 to 0.715
Left middle frontal gyrus	0.289	0.265	−0.162 to 0.638
Left insula	0.376	0.075	−0.035 to 0.701
PCC	0.321	0.208	−0.125 to 0.657

PCC, posterior cingulate cortex.

Perimenopausal participants were stratified by the CV of E2 (median CV=0.89) into:

High-fluctuation subgroup (CV>0.85, n=8): E2 levels showed extreme variability (e.g., 10–59 pg/mol).

Low-fluctuation subgroup (CV≤0.85, n=8): E2 levels were relatively stable (e.g., 12–19 pg/mol).

Independent samples t-tests showed:

Significantly higher RMFG connectivity in the high-fluctuation subgroup (1.23 ± 0.38) than in the low-fluctuation subgroup (0.79 ± 0.32; t = −2.24, p=0.043). A marginally significant increase in LIL connectivity in the high-fluctuation subgroup (0.18 ± 0.19) vs. the low-fluctuation subgroup (0.01 ± 0.20; t = −1.98, p=0.068; Table 5).

**Table 5:** DMN connectivity in high vs. low E2 fluctuation subgroups (perimenopausal group).

Brain region	High-fluctuation (n=8) (mean ± SD)	Low-fluctuation (n=8) (mean ± SD)	t-Value	p-Value
Right middle frontal gyrus	1.23 ± 0.38	0.79 ± 0.32	−2.24	0.043
Left middle frontal gyrus	0.48 ± 0.29	0.38 ± 0.26	−0.81	0.432
Left insula	0.18 ± 0.19	0.01 ± 0.20	−1.98	0.068
PCC	0.92 ± 0.41	0.76 ± 0.47	−0.89	0.387

PCC, posterior cingulate cortex.

### Confounder control

#### Cognitive status as a confounder

To rule out cognitive function as a confounder, we performed two-sample t-tests with DMN connectivity as the dependent variable, group as the independent variable, and Stroop effect size as an additional covariate. The enhanced connectivity in the four key DMN regions remained significant (all p<0.05 after AlphaSim correction), indicating DMN changes are independent of cognitive performance.

Post-hoc correlations between DMN connectivity and Stroop task performance revealed partial functional links: connectivity of the right middle frontal gyrus and PCC was significantly correlated with Stroop task metrics (right middle frontal gyrus vs. Stroop effect size: r=0.382, p=0.035; PCC vs. Stroop incongruent accuracy: r=0.367, p=0.042), while no significant correlations were observed for the left middle frontal gyrus or left insula (all p>0.05). This suggests DMN reorganization partially supports cognitive stability but is not driven by cognitive differences.

#### Depressive symptoms as a confounder

To rule out depressive symptoms as a confounder, we repeated the two-sample t-tests with PHQ-9 scores added as a covariate. Sensitivity analyses further confirmed this:

Excluding the premenopausal participant with moderate depressive symptoms (PHQ-9 ≥10) did not change core findings;

Stratified analysis excluding participants with subclinical or moderate depressive symptoms (n=6: 2 perimenopausal, 4 premenopausal) replicated the core result – perimenopausal women still exhibited enhanced DMN connectivity in the four key regions (all p<0.05 after AlphaSim correction);

Restricting analysis to the “minimal symptoms” subgroup (PHQ-9 0–4, n=25: 14 perimenopausal, 11 premenopausal) also confirmed the enhanced DMN connectivity pattern (all p<0.05 after AlphaSim correction).

These results confirm subclinical mood differences do not drive DMN changes.

#### Motion and education as confounders

Motion control: No participant had a mean framewise displacement (FD) exceeding the threshold for motion contamination (>0.5 mm). Excluding frames with FD >0.2 mm (2.3 % of total frames) did not alter DMN connectivity results, and mean FD did not differ between groups (perimenopausal: 0.18 ± 0.06 mm vs. premenopausal: 0.16 ± 0.05 mm, p=0.321), ruling out motion as a confounder (Supplementary Table S5).

Education control: Subgroup analyses by education level (<12 years vs. ≥12 years) confirmed education does not confound results: the perimenopausal group showed significant DMN connectivity enhancement in the ≥12 years subgroup (n=22: 10 perimenopausal, 12 premenopausal; all p<0.05), while the <12 years subgroup (n=9) exhibited a similar trend (non-significant, likely due to small sample size) with no reversed effects. This aligns with the initial inclusion criterion of ≥12 years of education (to avoid floor effects in cognitive tests), confirming the criterion did not bias core findings.

### MRI data quality validation

To address potential concerns about the 6 min rsfMRI acquisition duration (vs. the ∼10 min consensus recommendation), two validation analyses were performed:

Temporal signal-to-noise ratio (tSNR): The mean tSNR in key DMN regions was 45.2 ± 8.7 (range: 32.1–58.9), exceeding the threshold of 30 for reliable resting-state connectivity estimates;

Time series subset analysis: Splitting the 180 vol time series into two 90 vol subsets (each 3 min) and re-running DMN connectivity analyses replicated the core finding: perimenopausal women showed enhanced connectivity in the four key regions (all p<0.05 after AlphaSim correction; Supplementary Table S6).

These results confirm the 6 min acquisition provided stable, high-quality data, ruling out acquisition duration as a confounding factor.

## Discussion

In the present study, we addressed the core research question by characterizing DMN functional connectivity differences between perimenopausal and premenopausal women and elucidating their associations with estrogen dynamics. We found that perimenopausal women exhibited significantly enhanced functional connectivity in the bilateral middle frontal gyri, left insula, and posterior cingulate cortex compared to premenopausal women, with concurrently elevated ALFF values in these regions. These differences were specifically associated with estrogen decline and fluctuation rather than other sex hormones, and remained independent of cognitive function, depressive symptoms, motion artifacts, and other confounders – providing support for our hypothesis that these changes reflect a compensatory mechanism to preserve cognitive and emotional stability during perimenopause.

### Core findings in context of perimenopausal hormonal physiology

The observed DMN functional reorganization (not impairment) is tightly linked to the hormonal hallmark of perimenopause: unstable and declining E2 levels. Notably, no significant changes were observed in the canonical MPFC core, highlighting the specificity of DMN reorganization to functional extension nodes (MFG) and pDMN core (PCC). Our results confirm E2 levels were significantly lower in perimenopausal women (19.19 ± 15.35 pg/mol) compared to premenopausal women (101.47 ± 70.34 pg/mol, p<0.001), consistent with prior reports of ovarian function decline during this transition [1], 3]. Dasgupta and Rehman [3] emphasized that perimenopausal estrogen secretion is characterized by “instability rather than steady decline” due to disrupted hypothalamic-pituitary-ovarian axis feedback – this physiological feature directly explains our nuanced hormone-brain findings: no significant linear correlations between E2 and the four key DMN regions in perimenopausal women (r=0.098–0.215, all p>0.05), while a significant positive correlation between E2 and LIL connectivity was observed in premenopausal women (r=0.489, Bonferroni-corrected p=0.032).

It is critical to clarify that the group-level enhancement of DMN connectivity and the absence of individual-level E2-DMN linear correlations are not contradictory. The former reflects a population-wide adaptive response to perimenopausal E2 decline [8], while the latter arises from E2 fluctuation disrupting linear hormone-brain coupling [3]. This interpretation is supported by two targeted supplementary analyses: (1) E2 decline magnitude correlated marginally significantly with right MFG (RMFG) (r=0.402, p=0.058) and PCC (r=0.376, p=0.075) compensatory magnitude; (2) perimenopausal women in the high-E2-fluctuation subgroup exhibited significantly higher RMFG connectivity (1.23 ± 0.38 vs. 0.79 ± 0.32, p=0.043) than the low-fluctuation subgroup. These results align with Ruehr et al.’s [8] systematic review, which highlighted that estrogen modulates brain networks via “dynamic rather than static mechanisms” during reproductive transitions.

Notably, other sex hormones (FSH, LH, progesterone, PRL) showed no significant associations with DMN connectivity (all p>0.05), confirming E2’s specific regulatory role. This is consistent with Mosconi et al. [6], who demonstrated that DMN regions (e.g., PCC, insula) are densely populated with estrogen receptors (ER-α/β), while gonadotropins like FSH exert minimal direct effects on brain network connectivity.

### Functional significance of enhanced DMN connectivity: a compensatory mechanism

The enhanced connectivity of the bilateral MFG, LIL, and PCC reflects a targeted compensatory strategy to maintain cognitive and emotional stability amid E2 decline, with each region contributing uniquely to this adaptation – supported by supplementary analyses linking E2 dynamics to region-specific compensation.

#### Bilateral middle frontal gyri: executive control compensation

The bilateral MFG – core nodes of the anterior DMN (aDMN) – exhibited robust connectivity enhancement, aligning with their role in executive function (cognitive inhibition, working memory) and high ER density [28], 29]. Greater E2 decline correlated with stronger RMFG compensation (marginally significant, p=0.058), suggesting the brain intensifies RMFG connectivity to offset prefrontal dysfunction induced by E2 loss. This is consistent with evidence that E2 modulates prefrontal synaptic plasticity by regulating dopamine and glutamate release [28] – disruptions in this process (e.g., perimenopausal E2 fluctuation) may weaken linear E2-MFG coupling but trigger compensatory connectivity.

High E2 fluctuation further amplified RMFG connectivity (p=0.043), a finding supported by observations that perimenopausal cognitive fluctuations are linked to prefrontal functional reorganization [29]. The lack of a linear E2-MFG correlation in perimenopausal women likely stems from this non-linear regulatory process – E2 dynamics (not absolute levels) drive connectivity changes. Post-hoc analyses further validate this: RMFG connectivity correlated with Stroop effect size (r=0.382, p=0.035), linking aDMN reorganization to preserved cognitive inhibition – consistent with findings that E2 therapy enhances prefrontal activation during cognitive tasks [30].

#### Posterior cingulate cortex (PCC): DMN hub preservation

As the core hub of the posterior DMN (pDMN), the PCC interacts with hippocampal and parietal regions to support episodic memory and attention [31]. Our supplementary analyses showed a trend toward positive correlation between E2 decline and PCC compensation (r=0.376, p=0.075), indicating the PCC prioritizes compensation to preserve memory function amid E2 loss. This aligns with PET studies demonstrating that E2 administration increases PCC metabolic activity in postmenopausal women [32] – confirming the PCC’s sensitivity to E2.

The PCC’s compensatory enhancement is critical for maintaining DMN integrity, as it is the only DMN node that interacts directly with nearly all other nodes [31]. Prior work emphasizes that PCC connectivity correlates with episodic memory performance [31], and our finding that PCC connectivity correlates with Stroop incongruent accuracy (r=0.367, p=0.042) further validates that PCC compensation supports sustained cognitive precision – even without linear E2-PCC coupling.

#### Left insula: emotional and interoceptive adaptation

The LIL – critical for integrating interoceptive signals and positive emotion regulation [33] – showed enhanced connectivity and a marginally significant association with E2 fluctuation (p=0.068). Perimenopausal women often experience emotional instability (e.g., mood swings) due to E2 oscillations [7], and LIL enhancement may reflect a neural effort to mobilize positive emotional resources and stabilize interoceptive awareness (e.g., mitigating hot flashes’ impact on brain function). This aligns with evidence that the left insula is specifically linked to positive emotion regulation [33] – an adaptive function during hormonal transition.

The premenopausal group’s significant E2-LIL correlation (r=0.489, p=0.032) further supports the LIL’s sensitivity to stable E2 levels. Prior observations note that stable E2 levels in premenopausal women maintain consistent hormone-brain coupling [7], while perimenopausal fluctuation disrupts this – shifting to a fluctuation-dependent compensatory mode. This explains why linear E2-LIL coupling is absent in perimenopausal women but present in premenopausal women.

### Interpreting non-significant E2-DMN linear correlations in perimenopausal women

The absence of linear E2-DMN correlations in perimenopausal women does not negate E2’s role in DMN reorganization but instead reflects the unique biology of perimenopause – specifically, E2’s “unstable decline” and the brain’s shift to non-linear compensation.

#### E2 fluctuation: the “missing link” in linear correlation models

Perimenopausal E2 levels exhibit a coefficient of variation (CV=0.89) nearly 1.3 times higher than premenopausal women (CV=0.69), introducing “biological noise” that disrupts linear coupling [3]. For example, two perimenopausal participants with identical low E2 (10 pg/mol) may show vastly different RMFG connectivity (0.71 vs. 1.26; raw data) – a phenomenon attributed to “neuroendocrine chaos” during perimenopause [1]. Linear correlation models assume consistent variable relationships, making them insufficient to capture this complexity [8].

Supplementary analyses resolve this gap: E2 decline/fluctuation directly drives compensation, even without linear coupling. This aligns with neurophysiological evidence that E2 modulates neural activity via multiple pathways (synaptic plasticity, neurotransmitter balance [28]) – not just linear receptor binding. Prior work further confirms that resting-state network changes during hormonal transitions are often non-linear, requiring dynamic analyses (e.g., E2 decline magnitude) rather than static linear models [21].

#### Non-linear compensation: an adaptive response to hormonal chaos

The shift from linear E2 regulation to non-linear compensation is a key adaptive feature of perimenopausal brain function. The brain uses a “dynamic compensation” strategy: strengthening RMFG/LIL connectivity in response to high E2 fluctuation (to stabilize executive/emotional function) and enhancing PCC/RMFG connectivity in response to greater E2 decline (to preserve memory/attention). This framework is supported by three lines of evidence: group-level DMN connectivity/ALFF enhancement in perimenopausal women (all p<0.05) [27]; supplementary analyses linking E2 dynamics to compensation (r=0.376–0.402) [8]; preserved cognitive/emotional function (Stroop task: p=0.495–0.519; PHQ-9: p=0.067) despite reduced E2 [7]. Together, these findings confirm that non-linear DMN compensation effectively mitigates the functional impact of E2 decline/fluctuation – extending observations from postmenopausal populations [32] to the perimenopausal phase.

### Current study’s contributions to perimenopausal brain function research

Our work advances perimenopausal brain function research by challenging linear hormone-brain models, demonstrating that perimenopausal E2 dynamics (not absolute levels) drive DMN reorganization – resolving the paradox of “E2 decline but DMN enhancement.” We strengthen methodological rigor by using dual multiple comparison corrections (AlphaSim + TFCE), confounder control (age, education, cognitive/mood/motion), and supplementary analyses to validate findings – addressing limitations of prior studies that ignored E2 fluctuation or confounders [32]. Additionally, we identify region-specific compensation, highlighting RMFG and LIL as key targets of E2 fluctuation-dependent compensation – providing a targeted foundation for future interventions. These findings extend observations of estrogen-related network changes in postmenopausal populations to the perimenopausal phase, reinforcing the notion that neural adaptation commences early in the hormonal transition [8], 27].

### Clinical implications for perimenopausal health management

The findings hold important clinical value for perimenopausal women’s health. First, RMFG and LIL connectivity, along with their responses to E2 fluctuation, may serve as objective neuroimaging biomarkers for evaluating perimenopausal brain health – complementing subjective scales (e.g., MRS) and detecting subtle neural changes before behavioral symptoms emerge. Second, interventions that stabilize E2 levels (e.g., personalized hormone therapy) or target DMN hubs (e.g., cognitive training for MFG/PCC) could mitigate cognitive/emotional symptoms by supporting compensatory mechanisms. For women unable or unwilling to use hormone therapy, non-pharmacological approaches targeting prefrontal-PCC-insula networks (e.g., mindfulness-based stress reduction) may also enhance compensatory capacity. Third, future longitudinal studies could explore whether reduced DMN compensatory capacity (e.g., low RMFG response to E2 decline) predicts increased risk of postmenopausal cognitive decline or depression – enabling early identification of high-risk individuals and timely intervention.

### Study limitations and future directions

Despite rigorous design, this study has several limitations. The small sample size (n=31) limits the generalizability of trend-level findings (e.g., E2-DMN correlations) and may have reduced statistical power to detect weak linear associations. Future studies with larger cohorts are needed to validate these trends. Additionally, outlier sensitivity analysis (MAD method) was performed, and excluding 2 outliers did not alter core DMN results (all p<0.05 after AlphaSim correction), enhancing robustness, though post-hoc power analysis confirmed 89 % power for primary DMN connectivity outcomes (Supplementary Table S2). The cross-sectional design cannot establish causality; longitudinal studies tracking perimenopausal progression (early → late) are needed to confirm E2 dynamics drive DMN compensation. We did not stratify by perimenopausal symptom severity (e.g., mild vs. severe hot flashes), which may modulate compensation magnitude – an important direction for future research. While dual AlphaSim + TFCE correction enhances reliability, family-wise error (FWE) correction (more stringent for large samples) was not used due to small sample size, which should be addressed in larger-scale replications.

Notably, the study further lacks characterization of socioeconomic status (e.g., income, occupation, social support) and genetic polymorphisms (e.g., estrogen receptor ER-α/β gene variants, APOE genotype), which are key modifiers of brain network plasticity and neural sensitivity to estrogen decline, limiting the exploration of gene-environment-hormone interactions. We also did not collect data on mild, well-controlled comorbidities (e.g., mild dyslipidemia, thyroid dysfunction), prior non-hormonal medical interventions, lifestyle factors (e.g., physical activity, sleep quality, diet, smoking/alcohol consumption) and reproductive history (e.g., parity, breastfeeding duration), all of which may subtly modulate neural activity and DMN functional connectivity as unmeasured confounders. Additionally, cognitive assessment was limited to the Stroop task for executive function, with no evaluations of other DMN-related cognitive domains (e.g., episodic memory, working memory, sustained attention), restricting comprehensive characterization of the behavioral correlates of DMN reorganization.

Future research should address these gaps by conducting longitudinal follow-up to assess whether DMN connectivity predicts long-term cognitive/emotional outcomes, exploring how interventions (hormone therapy, behavioral approaches) modulate DMN function, integrating structural MRI (gray matter volume, diffusion tensor imaging) to investigate “structure-function” relationships, and including additional cognitive tasks (e.g., episodic memory, sustained attention) to better characterize behavioral correlates of DMN reorganization. Moreover, future studies should recruit larger, more diverse cohorts with detailed collection of socioeconomic, genetic, lifestyle and reproductive history data, stratify participants by perimenopausal symptom severity and duration, and apply more stringent statistical correction methods (e.g., FWE) to validate the core findings. Investigations into gene-environment-hormone interactions and the modulation effects of comorbidities on DMN compensation will also help to refine the understanding of perimenopausal brain plasticity. These steps will further refine our understanding of perimenopausal brain adaptation and inform evidence-based strategies for supporting women’s brain health during this critical transition.

## Conclusions

In conclusion, perimenopausal women with unstable and decreased E2 levels show significant functional reorganization of the DMN, characterized by enhanced functional connectivity and elevated ALFF values in the bilateral MFG, LIL, and PCC. These changes are specifically associated with E2 dynamics (decline and fluctuation) and independent of other sex hormones, cognitive function, depressive symptoms, or motion artifacts – suggesting a potential compensatory neural mechanism to maintain cognitive and emotional stability amid E2 decline. The absence of linear E2-DMN correlations in perimenopausal women reflects non-linear compensation driven by E2 fluctuation, while the premenopausal E2-LIL correlation confirms stable E2 levels support linear hormone-brain coupling.

Given the small sample size (n=31) of this cross-sectional study, the above findings are preliminary and their generalizability is limited, which precludes definitive causal inferences between E2 dynamics and DMN reorganization and adequate judgement of the clinical utility of the identified DMN subregions as biomarkers for perimenopausal brain health. This study provides preliminary targeted neuroimaging evidence for E2-mediated brain function adaptations during the perimenopausal transition, and the identified DMN subregions (especially RMFG and LIL) may serve as candidate biomarkers for evaluating perimenopausal brain health. Further large-scale, longitudinal investigations with diverse cohorts are urgently warranted to validate these findings, clarify the temporal dynamics and causality of E2-DMN interactions, explore the modulation effects of socioeconomic, genetic and lifestyle factors on DMN compensatory capacity, and assess the long-term predictive value of these DMN changes for cognitive and emotional health in perimenopausal and postmenopausal women.

## Supplementary Material

Supplementary Material
