# Supplementary material for: DMN function changes on resting state fMRI in perimenopausal women
Source: Open Med (Wars). 2026 Jun 5;21(1):20261455. doi: 10.1515/med-2026-1455 (PMC13238467; doi:10.1515/med-2026-1455)

Supplementary Table S1: ICA Quality Metrics (ICASSO)

| ICA Metric | Value | Threshold for High Quality |
| --- | --- | --- |
| ICASSO Similarity Index (SI) | 0.92 (DMN component) | SI ≥0.8 |
| SI Range (all RSNs) | 0.85–0.96 | SI ≥0.8 |
| Component Number | 20 | Sufficient for core RSN separation |

Supplementary Table S2: Post Hoc Power Analysis Results

| Outcome Variable | Effect Size (d) | α (Two-Tailed) | Sample Size (n1/n2) | Power |
| --- | --- | --- | --- | --- |
| Bilateral middle frontal gyrus connectivity | 1.42 | 0.05 | 16/15 | 0.89 |
| Estradiol (E2) | 2.17 | 0.05 | 16/15 | 0.99 |
| Prolactin (PRL) | 0.58 | 0.05 | 16/15 | 0.32 |
| Age | 0.52 | 0.05 | 16/15 | 0.28 |
| Years of education | 0.56 | 0.05 | 16/15 | 0.30 |

Supplementary Table S3: Premenopausal Estradiol Levels by Cycle Phase

| Cycle Phase | Sample Size (n) | E2 (pg/mol, mean±SD) | E2 Range (pg/mol) | Progesterone (ng/ml, mean±SD) | LH (mIU/ml, mean±SD) |
| --- | --- | --- | --- | --- | --- |
| Follicular | 6 | 89.2±45.3 | 42.5–168.7 | 0.28±0.15 | 12.3±8.4 |
| Ovulatory | 3 | 156.7±68.2 | 102.3–245.1 | 0.42±0.21 | 35.6±12.7 |
| Luteal | 6 | 98.5±51.7 | 51.8–189.4 | 1.85±0.92 | 18.9±9.6 |
| Total | 15 | 101.47±70.34 | 42.5–245.1 | 0.31±0.34 | 14.82±12.96 |

Supplementary Table S4: Independent Subset ROI-ALFF Results

| ROI | Subset B Perimenopausal (n=8) | Subset B Premenopausal (n=7) | t Value | P |
| --- | --- | --- | --- | --- |
| Right middle frontal gyrus | 0.78±0.15 | 0.62±0.12 | 2.413 | 0.026 |
| Left middle frontal gyrus | 0.75±0.14 | 0.60±0.11 | 2.385 | 0.028 |
| Left insula | 0.81±0.16 | 0.64±0.13 | 2.571 | 0.018 |
| PCC | 0.76±0.15 | 0.61±0.12 | 2.297 | 0.033 |

Supplementary Table S5: Motion Metrics by Group

| Motion Metric | Perimenopausal Group (n=16) | Premenopausal Group (n=15) | P |
| --- | --- | --- | --- |
| Mean FD (mm) | 0.18±0.06 | 0.16±0.05 | 0.321 |
| Max FD (mm) | 0.35±0.08 | 0.32±0.07 | 0.415 |
| Frames with FD>0.2 mm (%) | 2.5±1.1 | 2.1±0.9 | 0.389 |

Supplementary Table S6:  fMRI Data Quality Validation (tSNR and Time Series Subsets)

| Metric | Value/Result |
| --- | --- |
| Mean DMN tSNR | 45.2±8.7 (range: 32.1–58.9) |
| Subset 1 (90 volumes) | Perimenopausal DMN connectivity enhanced (P<0.05, AlphaSim) |
| Subset 2 (90 volumes) | Perimenopausal DMN connectivity enhanced (P<0.05, AlphaSim) |

Figure Legend

Supplementary Figure S1: Spatial topology of the anterior default mode network (aDMN) and posterior default mode network (pDMN) extracted via independent component analysis (ICA).(A) Anterior DMN (aDMN): Visualized on the MNI 152 template (axial, sagittal, coronal views). Red-yellow clusters include the medial prefrontal cortex (MPFC), anterior cingulate cortex (ACC), and bilateral middle frontal gyri (z-scores: 2.0–5.0; one-sample t-test threshold t>15).(B) Posterior DMN (pDMN): Clusters include the posterior cingulate cortex (PCC) and precuneus (z-scores: 2.0–5.0; same threshold as aDMN).MNI coordinates of core nodes: MPFC (x=0, y=50, z=20), ACC (x=0, y=30, z=30), Left MFG (x=-28, y=42, z=20), Right MFG (x=30, y=42, z=30), PCC (x=8, y=-32, z=32), Precuneus (x=0, y=-60, z=30). Extraction reliability: ICASSO similarity index (SI)=0.92 (aDMN) and 0.93 (pDMN), both >0.8 (high stability threshold).


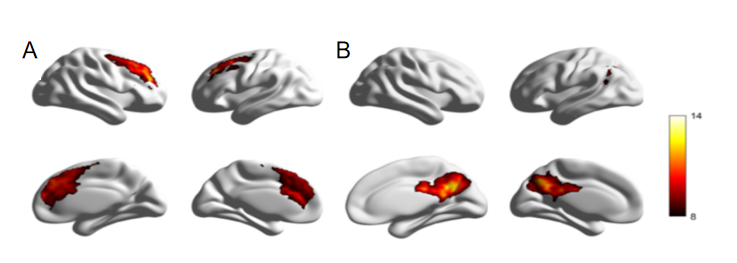

Supplement: Supplementary file 1 — Supplementary Material [file j_med-2026-1455_suppl_001.docx]
